# Validating scoring systems for fracture healing in infants and young children: pilot study

**DOI:** 10.1007/s00247-021-05038-3

**Published:** 2021-04-13

**Authors:** Samuel Crompton, Fabrizio Messina, Gillian Klafkowski, Christine Hall, Amaka C. Offiah

**Affiliations:** 1 The University of Sheffield, Western Bank, Sheffield, South Yorkshire S10 2TN UK; 2 Bayer Pharmaceutical PLC, Data Science UK, Berkshire, Reading UK; 3 Radiology Department, Sheffield Children’s Hospital, Western Bank, Sheffield, South Yorkshire UK; 4 Great Ormond Street Hospital for Children NHS Foundation Trust, London, UK

**Keywords:** Femur, Fracture, Fracture healing, Infant, Radiography, Review

## Abstract

**Background:**

Recent studies have analysed birth-related clavicular fractures to propose time frames for healing that could be applied to dating of all fractures in cases of suspected child abuse.

**Objective:**

To assess differences in healing rates between femoral fractures and birth-related clavicular fractures in infants and young children.

**Materials and methods:**

A retrospective 5-year pilot study of femoral fractures in children younger than 3 years of age was performed. Anonymised radiographs were independently scored by two radiologists for stages of fracture healing. In cases of reader disagreement, radiographs were independently scored by a third radiologist.

**Results:**

In total, 74 radiographs (30 children) met the inclusion criteria. Fracture healing evolved over time with subperiosteal new bone formation (SPNBF) appearing first, followed by callus then remodelling. A power calculation for a single proportion, with a level of confidence of 95% and a margin of error of 5%, showed that in a definitive study, 359 radiographs would be required.

**Conclusion:**

Although the overall pattern of healing is similar, in this small pilot study, the earliest times for SPNBF and callus formation in femoral fractures appeared to lag behind healing of birth-related clavicular fractures. Remodelling appeared earlier than remodelling of clavicular fractures. A power calculation has determined numbers of femoral radiographs (359) required for a definitive study.

## Introduction

Child abuse is estimated to affect 55 million children each year in Europe [1], with skeletal injuries being a common finding [2, 3]. Radiologic imaging is therefore key in diagnosis [4]. Radiologists must interpret radiographs to identify fracture location and type, which, in turn, suggest the possible mechanism of injury [3]. In cases of suspected child abuse, histories are often inaccurate or absent, and injuries unwitnessed. Therefore, it may be difficult to accurately define when a fracture has occurred, in which situation radiologic dating is required to either confirm or refute the account of when the injury occurred and thus identify or eliminate potential perpetrators [5, 6]. It is accepted that time frames for stages of fracture healing change with age [7, 8]. There are only a few studies that analyse fractures in children younger than 3 years old, despite the fact that children in this age range are the most likely to be victims of physical abuse. Dating in this age group is largely based on the personal experience of the reporting radiologist or of authors of textbooks in which some guidance on dating is given based on very little primary research [7, 9–13]. Aiming to avoid pitfalls such as uncertainty over the true fracture date and obscuration of radiologic features by the presence of plaster cast, recent works from Walters et al. [7] and Fadell et al. [10] analysed radiographs of birth-related clavicular fractures to identify time frames for stages of fracture healing that could then be applied to the dating of any shaft fractures in cases of suspected child abuse [7, 10]. Although these time frames are useful, one study has shown that the radius heals faster than the tibia [14]. This suggestion that different bones heal at different rates warrants further investigation. Therefore, our aim was to test the hypothesis that time frames given for healing of birth-related clavicular fractures are applicable to accidental diaphyseal femoral fractures in children younger than 3 years of age. Our study seeks to address the implied suggestion of both Walters et al. [7] and Fadell et al. [10] that their criteria should be assessed in older infants and young children and in bones other than the clavicle. We propose that if each of the parameters identified by Walters et al. [7] and Fadell et al. [10] is met by 80% of the femoral fractures we have studied, then the hypothesis can be accepted. If the criteria for the clavicle are accurate for the femur (the largest bone in the body) and for older infants and children, then the criteria are likely to apply to the healing of all long bone (and possibly rib) shaft fractures in children up to 3 years old, although definitive studies of the other long bones might still be prudent. The number of radiographs required for a definitive study of the femur is unknown, hence this pilot study.

## Materials and methods

This is a retrospective review of all radiographs of fractured femurs in children younger than 3 years of age who were seen at Sheffield Children’s Hospital over a 5-year period from Mar. 1, 2011, to Feb. 29, 2016.

A search was performed on the hospital’s picture archiving and communications system to identify all radiographs of diaphyseal femoral fractures in children younger than 3 years old performed during the study period. Hospital notes available on the hospital’s electronic system were reviewed to identify the date of fracture occurrence, whether the fracture was accidental or inflicted (noted as accidental, probably accidental, probably inflicted or inflicted) and to identify fractures that might be pathological. Coding of accidental or inflicted was based on the note taking and opinions of clinicians who reviewed the patients on admission as well as whether any concerns of inflicted injury were raised and the outcomes, if any, of these concerns. Only fractures of known date of occurrence, where there was no suspicion of abuse noted (accidental) or discussions/meetings were held that deemed the injury more likely to be accidental (probably accidental) and where there was no underlying pathology were included.

All radiographs meeting the inclusion criteria were anonymised and scored independently by two paediatric radiologists (A.C.O., with 13 years of experience, and G.K., with 14 years of experience) for stages of fracture healing. A previous consensus trial read was performed on 10 radiographs not included in the study. In study cases for which there was disagreement between the two readers, radiographs were arbitrated by a third paediatric radiologist with more than 40 years’ of experience (C.H.), whose readings acted as the reference standard. Both anteroposterior (AP) and lateral projections were available to the readers.

Radiographs were scored for presence of subperiosteal new bone formation (SPNBF), callus and remodelling.

SPNBF is defined according to Walters et al. [7] as “new bone paralleling the original cortex of the bone with a linear configuration.” SPNBF was scored as present, probably present, probably not present, not present or present but indistinguishable from callus as used by Walters et al. [7]. In cases scored as ‘present but indistinguishable from callus’, readers were instructed to assess in terms of callus as was done by Walters et al. [7].

Callus was defined according to Walters et al. [7] as “mineralization first evident as amorphous opacity near the cortical margins of the fracture, and subsequently progressing centrifugally away from the injury site with a more spherical configuration.” Callus was scored as present, probably present, probably not present or not present.

Remodelling was defined as “completion of the acute healing phase; the fracture line is no longer discernible” [5]. Remodelling was scored as present, probably present, probably not present or not present.

For the purpose of data analysis, the scores present and probably present were condensed to present and the scores probably not present and not present were condensed to not present.

Data were analysed using the R software (version 3.4.1; R Foundation for Statistical Computing, Vienna, Austria). Interobserver reliability was calculated using kappa statistics. Based on the results of this pilot study, power calculations were performed for each radiographic parameter to identify the number of radiographs required for a definitive study.

Research ethics committee approval was not required for this study because there was no direct patient contact or involvement. Both health research authority and local research and development approvals were obtained.

## Results

In our initial search, 152 radiographs were identified, 78 of which were excluded for reasons summarised in Table 1. In cases where a patient was imaged more than once on the same day, only one set of radiographs were included in the study, since there would be no change in radiographic features of healing. A total of 74 radiographs (30 children; age range: 1–33 months, mean: 19 months) met the inclusion criteria, with the age range of the fractures being 0 to 198 days (mean: 16 days). When calculating kappa statistics, 14 reads were removed for the following reasons: obscured by cast (observer 1=3, observer 2=6) and missing data (observer 1=1, observer 2=4).

**Table 1:** Breakdown of radiographs excluded from this study

**Reason for exclusion**	**Number of radiographs**
No patient notes available	28
Pathological fracture	24
Inflicted injury	17
Date of fracture unidentifiable	3
Patient with two studies on the same day	3
No fracture identified	2
Patient 3 years of age or older	1

Table 2 shows a breakdown of the number of radiographs per patient and patient age.

**Table 2:** Breakdown of the number of radiographs included per patient and patient age

**Number of radiographs**	**Number of patients**	**Age (months) of patients at initial radiograph,** **median (range)**
5	1	25 (not applicable)
4	3	23 (10–23)
3	10	26 (9–31)
2	11	23 (3–33)
1	5	11 (1–24)

**Fig. 1:**
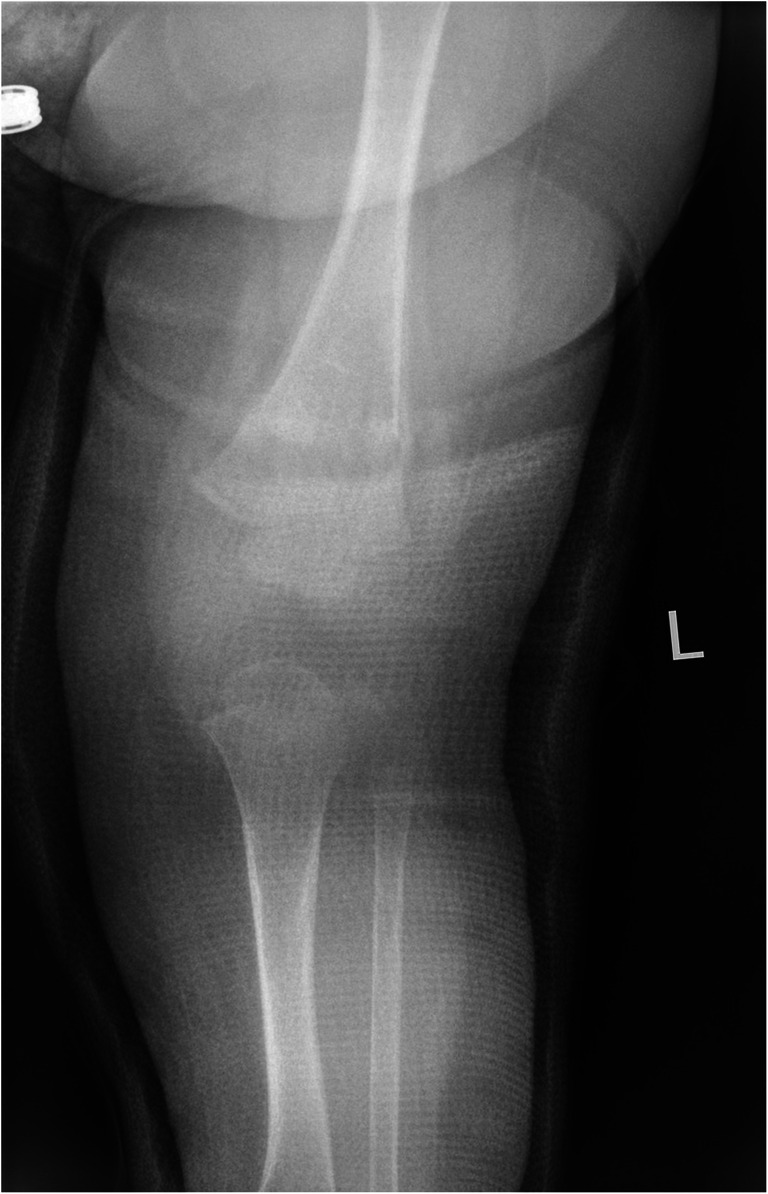
An anteroposterior radiograph of the left femur in an 8-month-old boy shows subperiosteal new bone formation

SPNBF was present on 27 of 73 radiographs (37.0%, 95% confidence interval [CI] 25.9–48.1%) (Fig. 1). In one case, SPNBF was scored as present but indistinguishable from callus and was therefore analysed in terms of callus rather than SPNBF (an approach also taken by Walters et al. [7]) and hence removed from the SPNBF results. Reader agreement between the two primary readers was moderate (kappa 0.452). The age range of fractures with SPNBF identified as present was 7–59 days. In fractures 0–6 days old, SPNBF was scored as present in 0 of 34 images. For those fractures ages 7–11 days, only 2 of 9 (22.2%, 95% CI 0–49.4%) were scored as SPNBF present. In fractures 12 days and older, SPNBF was scored as not present in 5 cases, these being 13, 14, 15, 34 and 198 days old. These five cases were all noted to be oblique/spiral fractures.

**Fig. 2:**
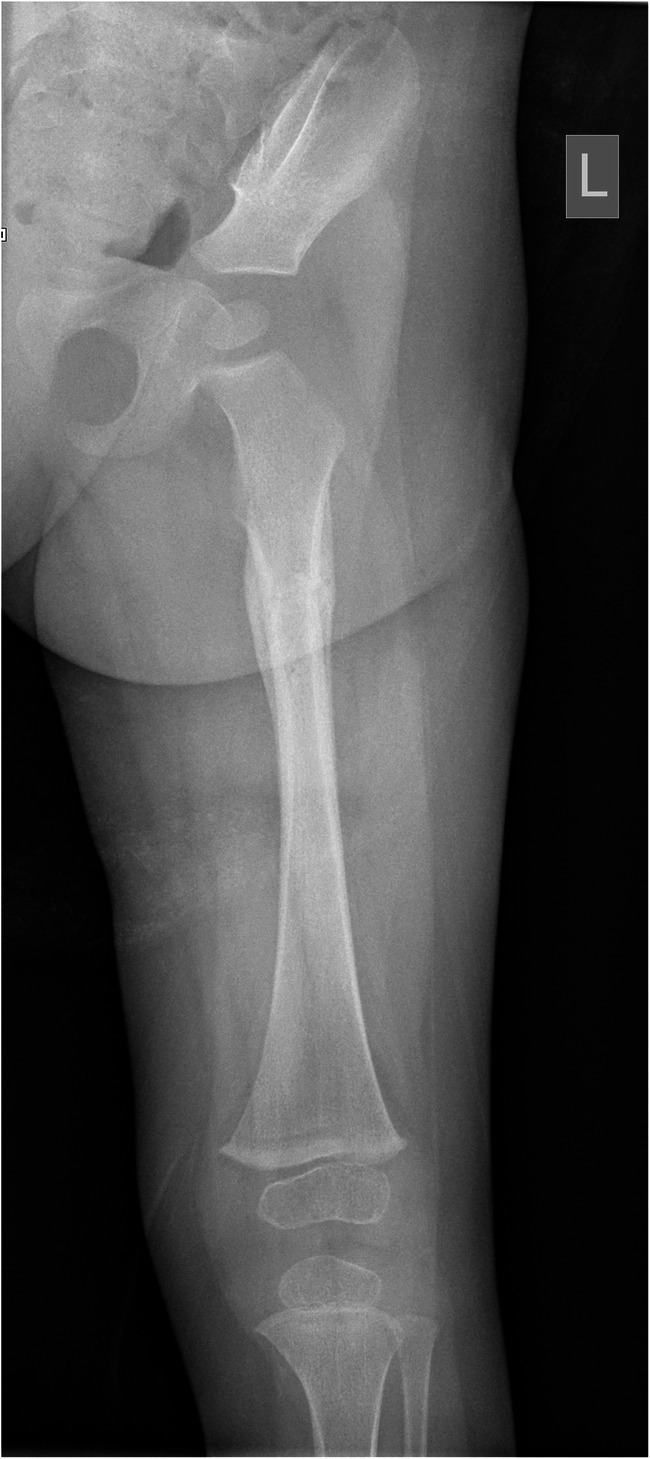
An anteroposterior radiograph of the left femur in a 15-month-old girl shows callus

Callus was identified as present on 21 of 74 radiographs (28.4%, 95% CI 18.1–38.7%) (Fig. 2). Reader agreement between the two primary readers was good (kappa 0.754). The age range of fractures with callus identified as present was 15–198 days. In fractures ages 15–26 days, callus was scored as present in 4/8 cases (50.0%, 95% CI 15.4–84.7%). In fractures 27 days and older, callus was scored as present in 17/19 cases (89.5%, 95% CI 75.7–100%); in the remaining 2 cases scored as callus not present, SPNBF was scored as present in both, while remodelling was scored as present in only 1.

**Fig. 3:**
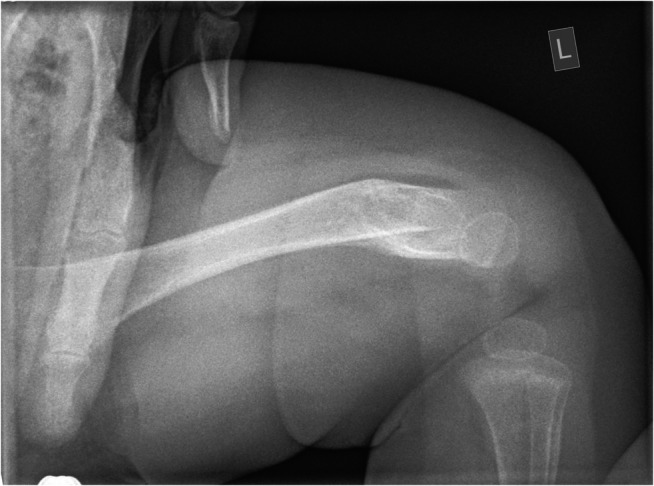
A lateral radiograph of the left femur in a 7-month-old girl shows remodelling

Remodelling was identified as present on 10 of 74 radiographs (13.5%, 95% CI 5.7–21.3%) (Fig. 3). Reader agreement between the two primary readers was moderate (kappa 0.527). The age range of fractures with remodelling identified as present was 26–198 days. Remodelling was scored as present in 4/15 fractures (26.7%, 95% CI 4.3–49.1%) between 26 and 37 days old. All 6 fractures ages 42 days and older were scored as present for remodelling.

The percentage of fractures for given fracture age ranges when each parameter was scored as present is summarised in Table 3.

**Table 3:** Percentage of fractures showing features of fracture healing

Feature of healing	Age of fracture	% of fractures showing feature (95% confidence interval)	Number of study radiographs
Subperiosteal new bone formation	0–6 days	0 (0–0)	34
	7–11 days	22.2 (0–49.4)	9
	12 days and older	83.3 (70.0–96.7)	30
Callus	0–14 days	0 (0–0)	47
	15–26 days	50.0 (15.4–84.7)	8
	27 days and older	89.5 (75.7–100)	19
Remodelling	0–22 days	0 (0–0)	53
	26–37 days	26.7 (4.3–49.1)	15
	42 days and older	100 (100–100)	6

Of the 74 fractures, 15 were noted to be impacted, 43 were oblique/spiral and 31 were transverse. Of the impacted fractures, 12 (80%) were 0–8 days old and all scored as not present for SPNBF. The remaining 3 cases (20%) were 22, 26 and 29 days old, and were all scored as present for SPNBF.

Of the 7 fractures 11–15 days old, there were 4 (57%) in which SPNBF, callus and remodelling were all scored as absent, 3 were oblique/spiral and 1 was transverse. In fractures 13–198 days old, SPNBF was scored as not present in 5 (17%). All of these were oblique/spiral fractures. The earliest SPNBF was seen for an oblique/spiral fracture was 7 days.

Tables 4, 5 and 6 compare our results with those of Walters et al. [7] and Fadell et al. [10]. There were four parameters identified by Walters et al. [7] and five identified by Fadell et al. [10] to which our study data could be compared. Only 4 of these 9 parameters had 80% of the femoral fractures we studied meeting the parameter.

**Table 4:** Comparison of our data with the findings of Walters et al. [7] for the presence of subperiosteal new bone formation (SPNBF)

Fracture age	SPNBF present (%)		SPNBF absent (%)		Reader disagreement (%)	
	Walters et al. [7]	Present study	Walters et al. [7]	Present study	Walters et al. [7]	Present study
0–7 days	3	3	92	97	5	–
8–9 days	29	20	43	80	28	–
≥10 days	98	78	2	22	–	–

**Table 5:** Comparison of our data with the findings of Walters et al. [7] for the presence of callus

Fracture age	Callus present (%)		Callus absent (%)		Reader disagreement (%)	
	Walters et al. [7]	Present study	Walters et al. [7]	Present study	Walters et al. [7]	Present study
0–8 days	0	0	100	100	0	–
9–14 days	35	0	38	100	27	–
≥15 days	98	78	2	22	0	–

**Table 6:** Comparison of our findings with the time line of fracture healing given by Fadell et al. [10]

**Fadell et al.** **[**10**]’s time line**	**% study data meeting time line**
Periosteal reaction unlikely <7 days old	100% (34/34)
If periosteal reaction is present alone, likely <11 days old	25% (2/8)
If callus and periosteal reaction are present, likely 11–49 days old	94% (17/18)
If remodelling is present, likely ≥5 weeks	70% (7/10)
Periosteal reaction unlikely beyond 7 weeks	33% (1/3)

A subanalysis compared reader agreement in and out of cast for each of the stages of fracture healing (Table 7).

**Table 7:** Reader agreement between the two primary readers for each of the radiographic parameters

**Radiographic feature**	**Reader agreement (Kappa)**		
	**Radiographs**		
	**All radiographs**	**No cast**	**Cast**
SPNBF	0.452	0.509	0.355
Callus	0.754	0.771	0.727
Remodelling	0.527	0.553	–

– not able to compute due to one reader scoring remodelling as present in zero cases, *SPNBF* subperiosteal new bone formation

Based on the data collected in this study, power calculations for a single proportion with a level of confidence of 95% and a margin of error of 5% showed that in a definitive study comparing femoral fractures with birth-related clavicular fractures for each of the three radiographic parameters (SPNBF, callus and remodelling), we would require 359, 310 and 186 radiographs, respectively.

## Discussion

Only a small number of studies have assessed the healing pattern of fractures in children younger than 3 years old. Although not based on published evidence, rates of fracture healing are said to vary with age, healing faster in younger age groups [7, 9]. Given that children in the youngest age group (younger than 3 years of age) are the most likely to be victims of abuse [7, 9–12], it is evident that this is an area requiring further research, particularly given the suggestion that different bones heal at different rates [14], which forms the focus of this pilot study. Our results agree with previous studies on birth-related clavicular fractures that fracture healing follows a predictable pattern, with SPNBF appearing first, followed by callus then remodelling. The SPNBF and callus stages of healing in femoral fractures appear to lag behind healing of birth-related clavicular fractures. Remodelling may be evident earlier in femoral fractures than in birth-related clavicular fractures, although the reason for this is unclear. Future studies should record the degree of fracture displacement and angulation as this may influence the onset of remodelling, as may the fact that femoral fractures are splinted/cast, whereas clavicular fractures are not.

Halliday et al. [9] examined long bone fractures in children ages 0–44 months. Although this study examined the age group in which inflicted injuries are most common, they examined a number of different bones and with relatively small numbers were unable to give ranges for, or compare between, specific bones. They stated that, “With the exception of SPNBF, the criteria relied on to date fractures are either not reproducible or are poor discriminators of fracture age” [9]. They concluded that the absence of SPNBF suggests that the fracture must be less than 11 days old. They scored all fractures 11 days and older positive for SPNBF apart from 1 fracture imaged at 37 and 78 days.

Yeo and Reed [8] examined femoral fractures in children ages 0–14 years. They focused on the callus stage of healing and included 25 patients in their study. Mean times for callus to appear, to bridge the fracture site and to mature were 11.7, 18.7 and 55.3 days, respectively.

Prosser et al. [15] examined accidental long bone fractures in children ages 0–6 years. The mean age was 4.8 years, thus a significant proportion of children in the study were not in the typical age range for physical abuse. The earliest appearance of periosteal reaction (SPNBF) was 5 days and they suggested that if periosteal reaction was present alone, the fracture was likely to be 5–14 days old. The presence of soft and hard callus ranged from 12–66 and 19–96 days, respectively. Remodelling was first seen at 45 days.

Warner et al. [16] studied features of healing in long bone fractures in children younger than 1 year old. They concluded that (1) if periosteal reaction is absent, then the fracture is likely to be less than 1 week old; (2) presence of periosteal reaction and callus indicate the fracture is at least 9–14 days old; (3) presence of bridging indicates the fracture is at least 2 weeks old; and (4) if remodelling is present, then the fracture is likely to be at least 51 days old. They included a relatively small number of radiographs (59) of various long bones and did not compare their healing rates.

Walters et al. [7] and Fadell et al. [10] recently assessed the stages of fracture healing in birth-related clavicular fractures. Walters et al. [7] concluded that SPNBF is highly unlikely in fractures less than 7 days old and is most often present by 10 days, and callus formation is highly unlikely in fractures less than 9 days old and most often present by 15 days. They did not assess fractures for remodelling [7]. Fadell et al. [10] concluded periosteal reaction is unlikely in fractures less than 7 days old and if periosteal reaction is present alone, the fracture is likely less than 11 days old. They suggest that if callus and periosteal reaction are both present, the fracture is likely between 11 and 49 day old; however, if bridging is also present in addition to callus and periosteal reaction, the fracture is likely between 20 and 63 days old. When remodelling is present, they suggest the fracture is likely 5 weeks or older. A limitation of the current study is that we did not assess fractures for bridging.

In comparison with the results of Warner et al. [16] and Halliday et al. [9], we found that in femoral fractures, the absence of SPNBF beyond 7 days was common and possible beyond 11 days; in fact, 26% of spiral/oblique femoral fractures between 13 and 198 days old did not demonstrate SPNBF. However, caution is required because the numbers in our pilot study are small and, because serial radiographs were not performed in every patient, it is possible that SPNBF occurred and resolved before later radiographs.

The appearance of callus formation in our study was later than that found in previous studies, which may be due to the fact that we were assessing only the femur (largest bone in the body). A further possible explanation for the delay of callus presentation in comparison with Warner et al. [16], who studied long bones, is that we studied children ages 3 years and younger, in comparison to children 1 year old and younger. Prosser et al. [15], whose cohort had a mean age of 4.8 years, found soft callus to be present in only 26% of fractures 15–21 days old and suggested that once hard callus or bridging appear the fracture is 3 weeks or older. The age of the child and the size of the bone appear to affect fracture healing.

Our findings suggest remodelling may appear earlier than found in previous studies, occurring earlier than 5 weeks in 3 of our cases. We postulate that this may be related to immobilisation in cast.

Healing patterns of birth-related clavicular fractures studied by Walters et al. [7] and Fadell et al. [10] are clearly reproduced in the femur. However, the general preliminary finding of this pilot study is that stages of fracture healing for birth-related clavicular fractures are not applicable to healing of femoral fractures (Tables 4, 5 and 6). Larger studies are required to confirm this.

Recent work by Drury and Cunningham [17] compared methods of dating fractures. Their findings suggest caution should be used when dating fractures with current published timetables. They highlight the need for further studies to identify accurate time frames for fracture healing in infants and young children [17]. Our results support this need.

Messer et al. [18] carried out a systematic review surrounding time lines for dating of paediatric fractures. They highlight the fact that previous research studies vary greatly with differing combinations of fracture sites and patient ages. They conclude further research is required that must assess the effect of fracture site and patient age on fracture healing time frames and also focus on validating the findings of previous studies. These suggestions are in keeping with the work carried out in our pilot study.

**Fig. 4:**
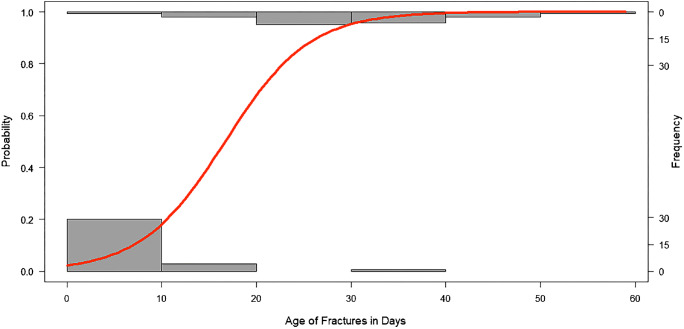
A logistic regression curve for the probability of subperiosteal new bone formation (SPNBF) in patients 12 months and older. The grey boxes correspond to frequency; the lower boxes indicate the frequency of SPNBF not present and the upper boxes indicate the frequency of SPNBF present

Figure 4 shows a logistic regression curve for the probability of SPNBF in patients ages 12 months and older. Numbers in the patient age group 11 months and younger were insufficient for a reliable regression curve to be produced. This should be investigated in a larger, definitive study.

**Fig. 5:**
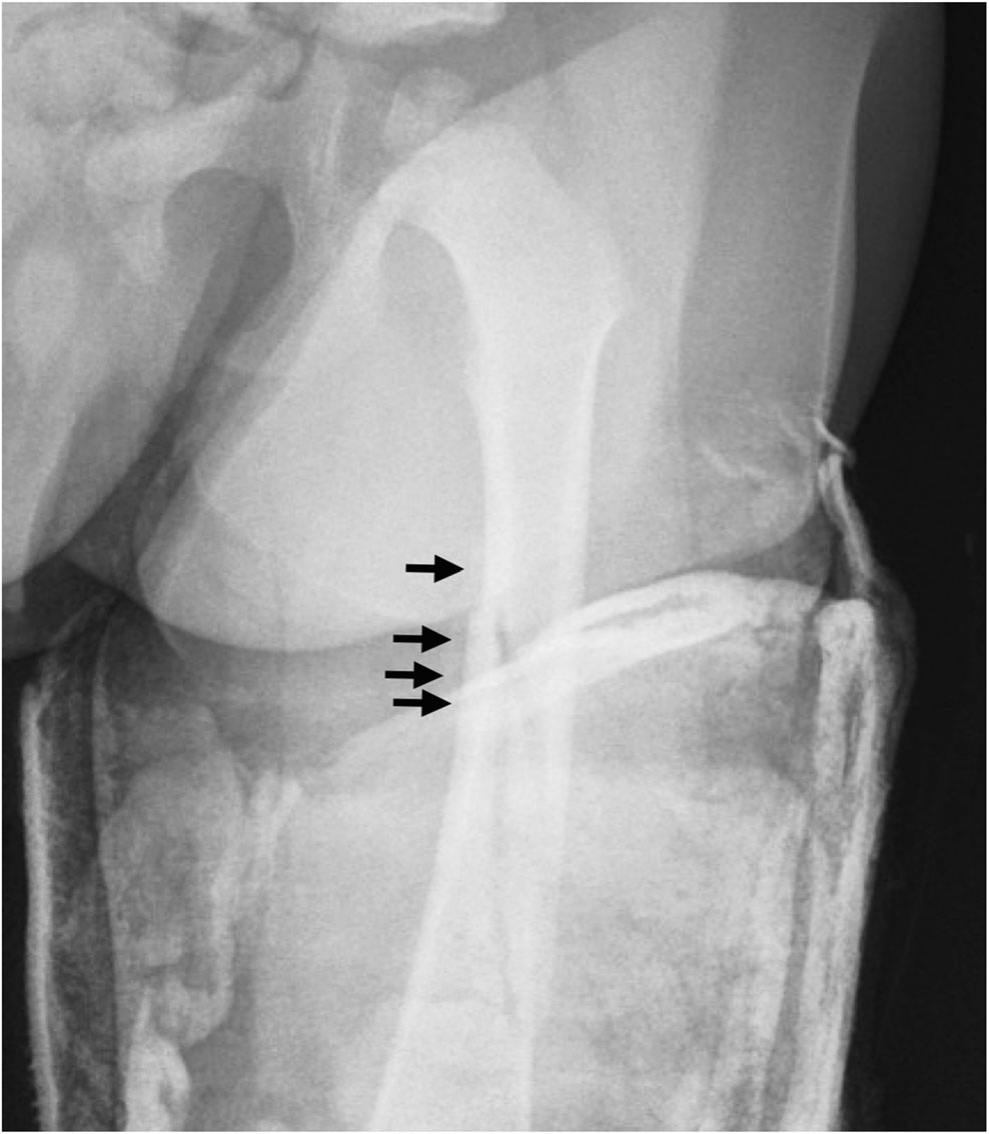
An anteroposterior radiograph of the left femur in a 22-month-old boy with the leg in a cast on the date of injury. The superior and medial edge of the cast (*arrows*) may easily be mistaken for subperiosteal new bone formation

Reader agreement in our study varied between the different stages of fracture healing, with callus showing substantial agreement and SPNBF and remodelling showing moderate agreement. Reader variability is an important consideration in the setting of inflicted injury and fracture dating. Reader variability may be due to reasons such as imaging through cast. In our subanalysis of reader agreement for fractures in and out of cast, we noted a moderate agreement for SPNBF out of cast compared with only a fair agreement in cast. Cast makes it more difficult to accurately assess the healing phase of the fracture and the edge of the cast may easily be mistaken for periosteal reaction (Fig. 5). It is also evident that there can be difficulty differentiating SPNBF from callus and we question the value of this differentiation.

Femoral fractures are most commonly immobilised with cast once diagnosed (as in 39% of fractures in this study). This is a limitation of the study because inflicted injuries may take longer to be diagnosed and therefore immobilisation may be delayed until after the healing process has begun. Immobilisation is known to improve fracture alignment; however, we were unable to find any clear evidence in the literature of its effect on fracture healing rate. The healing pattern and rate of inflicted fractures may not be the same as those of accidental fractures due to the (sometimes) delayed presentation of the former. This is a limitation of any study that seeks to determine accidental fracture healing patterns in order to use them to date inflicted fractures. However, paediatric radiologists are called upon to date fractures in cases of suspected abuse and, despite the limitations, studying accidental fractures is the only way of accruing sufficient data to propose time frames for fracture healing. On the other hand, a benefit of studying the femur is that the date of accidental fracture is usually very clear, leading to greater accuracy in identifying the actual age of a fracture. In comparison to Walters et al. [7] and Fadell et al. [10], who looked at clavicular fractures that are not immobilised, the stages in our study of femoral fractures appeared later, with the exception of remodelling. We postulate that immobilisation reduces the degree of periosteal reaction and callus, which may explain the absence of periosteal reaction by day 11 in some of our cases. We acknowledge that there is a lack of serial imaging of individual patients in our study; however, given that this is not routine in the clinical management of these patients, we could only use what radiographs were available. A final limitation is the small number of radiographs, although all of these radiographs were of a single bone (the femur).

In summary, we have looked at the femur, the largest bone in the body and a common site of inflicted injury, and compared the healing pattern and rate of healing of femoral fractures with those of birth-related clavicular fractures. It appears that while the overall pattern of healing is similar, the SPNBF and callus stages of femoral fracture healing in children up to the age of 3 years lag behind those of birth-related clavicular fractures. Caution is required when assessing SPNBF in oblique/spiral fractures and if the limb is in cast. Remodelling may first be apparent earlier than in the clavicle. It is evident that this is a complex field of study and that further larger studies are required to confirm these findings. Power calculations performed using the data from our study indicate that 359 femoral radiographs, ideally out of cast, would be required in a definitive study assessing SPNBF, callus and remodelling as parameters for fracture dating.

## Conclusion

Caution is required when using time frames of healing birth-related clavicular fractures to date femoral fractures (and possibly those of other bones) in children younger than 3 years of age. Further multicentre collaborative research is required in this area in order to accrue sufficient numbers of radiographs (out of cast) to define parameters for the accurate dating of suspected inflicted fractures of different types and affecting different bones.

## References

[1] World Health Organization (2018) European status report on preventing child maltreatment. http://www.euro.who.int/__data/assets/pdf_file/0017/381140/wh12-ecm-rep-eng.pdf?ua=1. Accessed 2 April 2019

[2] Dwek JR (2011). The radiographic approach to child abuse. Clin Orthop Relat Res.

[3] Offiah A, van Rijn RR, Perez-Rossello JM, Kleinman PK (2009). Skeletal imaging of child abuse (non-accidental injury). Pediatr Radiol.

[4] Kleinman PK (1998). Diagnostic imaging of child abuse.

[5] Offiah A, Hall C (2009). Radiological atlas of child abuse.

[6] Prosser I, Maguire S, Harrison SK (2005). How old is this fracture? Radiologic dating of fractures in children: a systematic review. AJR Am J Roentgenol.

[7] Walters MM, Forbes PW, Buonomo C, Kleinman PK (2014). Healing patterns of clavicular birth injuries as a guide to fracture dating in cases of possible infant abuse. Pediatr Radiol.

[8] Yeo LI, Reed MH (1994). Staging of healing of femoral fractures in children. Can Assoc Radiol J.

[9] Halliday KE, Broderick NJ, Somers JM, Hawkes R (2011). Dating fractures in infants. Clin Radiol.

[10] Fadell M, Miller A, Trefan L (2017). Radiological features of healing in newborn clavicular fractures. Eur Radiol.

[11] Akbarnia B, Torg JS, Kirkpatrick J, Sussman S (1974). Manifestations of the battered-child syndrome. J Bone Joint Surg Am.

[12] Kemp AM, Dunstan F, Harrison S (2008). Patterns of skeletal fractures in child abuse: systematic review. BMJ.

[13] Sanchez TR, Nguyen H, Palacios W (2013). Retrospective evaluation and dating of non-accidental rib fractures in infants. Clin Radiol.

[14] Malone CA, Sauer NJ, Fenton TW (2011). A radiographic assessment of pediatric fracture healing and time since injury. J Forensic Sci.

[15] Prosser I, Lawson Z, Evans A (2012). A timetable for the radiologic features of fracture healing in young children. AJR Am J Roentgenol.

[16] Warner C, Maguire S, Trefan L (2017). A study of radiological features of healing in long bone fractures among infants less than a year. Skeletal Radiol.

[17] Drury A, Cunningham C (2018). Determining when a fracture occurred: does the method matter? Analysis of the similarity of three different methods for estimating time since fracture of juvenile long bones. J Forensic Legal Med.

[18] Messer DL, Adler BH, Brink FW (2020). Radiographic timelines for pediatric healing fractures: a systematic review. Pediatr Radiol.

